# Mechanical Thrombectomy for Progressive Pulmonary Infarction in Intermediate-Risk Pulmonary Embolism

**DOI:** 10.7759/cureus.77611

**Published:** 2025-01-18

**Authors:** Jesse Liou, Hussein Kiliddar, Elias A Iliadis, Wissam Abouzgheib

**Affiliations:** 1 Division of Pulmonary Medicine, Cooper University Hospital, Camden, USA; 2 Division of Cardiology, Cooper University Hospital, Camden, USA

**Keywords:** hypoxia, pulmonary embolism, pulmonary infarct, pulmonary infarction, thrombectomy

## Abstract

Pulmonary infarction is commonly treated with therapy directed at the underlying etiology, a pulmonary embolism. Typically, anti-coagulation and supportive care are all that is needed. Advanced invasive therapy, such as catheter-directed thrombectomy, is a viable option, however, indications for this remain controversial. To our knowledge, we present the first case of a patient diagnosed with intermediate-risk pulmonary embolism, with no right ventricular (RV) dysfunction, that developed progressive pulmonary infarction and hypoxemia, and was successfully treated with aspiration thrombectomy. This highlights the need for consideration and the potential of mechanical thrombectomy, especially as proficiencies and technologies develop.

## Introduction

Pulmonary embolism (PE), along with its potentially life-threatening complications, such as pulmonary infarction, poses a significant risk to health. In the United States, the incidence ranges from 60-120 cases per 100,000 and 90-day mortality is as high as 20% [1]. Guidelines and risk stratification tools incorporate right ventricular (RV) dysfunction and cardiac biomarkers, such as Troponin and NT-BNP, to provide a framework for treatment [2]. Advanced therapies such as systemic thrombolysis, catheter-directed therapy (CDT) including thrombolysis or aspiration thrombectomy, and surgical embolectomy are generally reserved for hemodynamically unstable, also known as high-risk, pulmonary embolism [3]. Treatment of intermediate-risk pulmonary embolism is controversial, with conservative therapeutic anti-coagulation being the mainstay therapy. However, patients who continue to show decline may be considered for advanced therapeutics on an individual basis, but patient selection, timing, and optimal modality are not known. Catheter-directed therapies are effective at reducing pulmonary artery pressure, reversing right ventricular strain, and may be beneficial in those with impending hemodynamic collapse [4]. As patient selection for CDT revolves around a patient's hemodynamics, refractory hypoxia or pulmonary infarction, without hemodynamic collapse, has not been described as an indication for such treatment. We report on the first case of progressive pulmonary infarction, from an intermediate-risk pulmonary embolism, treated with catheter-directed aspiration thrombectomy.

## Case presentation

**Figure 1:**
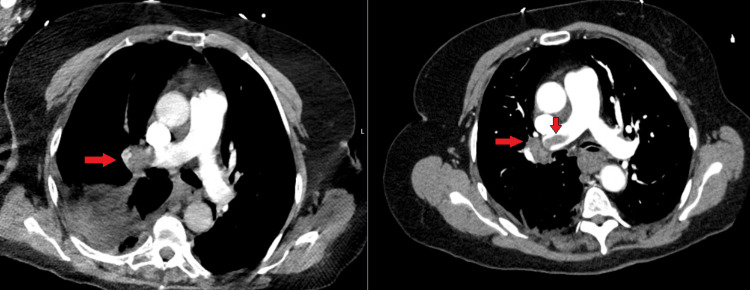
Pulmonary embolism on computed tomography angiography (CTA) chest CTA Chest (mediastinal windows) demonstrating right main pulmonary embolism (red arrows) in the emergency department (left) vs initial hospitalization (right).

**Figure 2:**
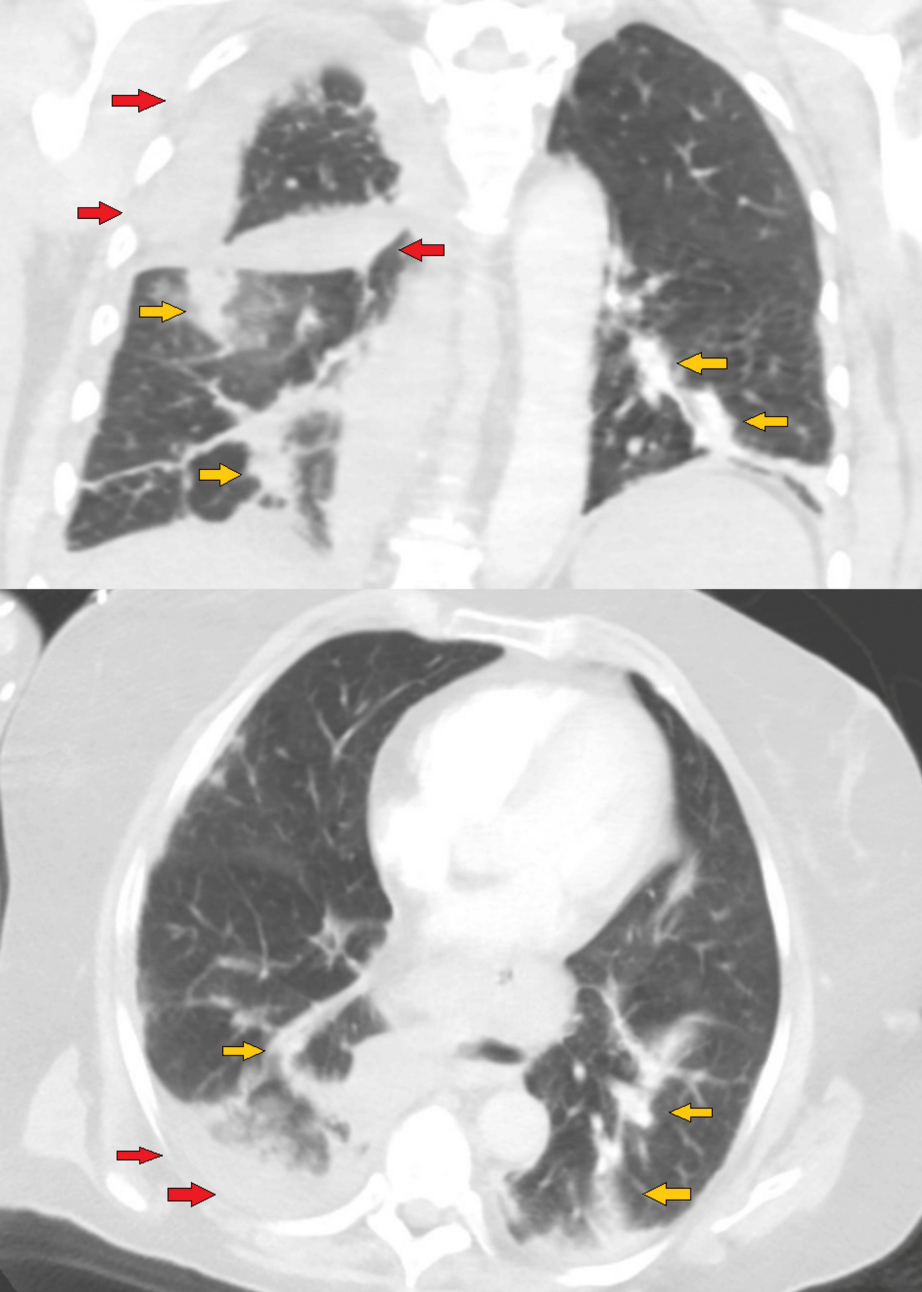
Pulmonary infarction & pleural effusions on computed tomography angiography (CTA) chest Emergency department CTA chest (lung windows) of coronal (top) and axial (bottom) cuts showing pulmonary infarct (yellow arrows) and pleural effusions (red arrows). Formal radiologist interpretation: "Extensive right pulmonary emboli. No CT evidence of right heart strain. Wedge-shaped consolidations in the right middle lobe and right lower lobe suspicious for developing infarct. Small right pleural effusion."

A 68-year-old woman with a past medical history of prior provoked deep vein thrombosis (DVT) in 2021 (completed anti-coagulation), stage III chronic kidney disease, and hypertension presented from a skilled nursing facility (SNF) for shortness of breath and hypoxemia. Initially, she required non-invasive ventilation with a fraction of inspired oxygen (FiO2) at 100% and was weaned to 6L nasal cannula (NC) in the emergency department. She was discharged just six days prior for a newly found intermediate-risk pulmonary embolism, with no right heart strain, in the right pulmonary artery. She was started on an apixaban load, and the SNF confirmed she was adherent to anti-coagulation. Relevant labs include a normal high-sensitivity Troponin at 8ng/L and a normal NT-PROBNP at 291pg/mL. Hemoglobin was at her baseline of 9.2g/dL. An electrocardiogram showed normal sinus rhythm, normal intervals, and no deviation of ST segments. A repeat chest computed tomography angiography (CTA) in the emergency department (ED) redemonstrated the pulmonary embolism in the right pulmonary artery, which was slightly improved (Figure 1). In addition, there was worsening consolidation, thought to be pulmonary infarct, and small pleural effusions (Figure 2). An echocardiogram was performed, which showed normal ejection fraction and no right heart strain or dysfunction. She was started Enoxaparin 1mg/kg and managed conservatively.

**Figure 3:**
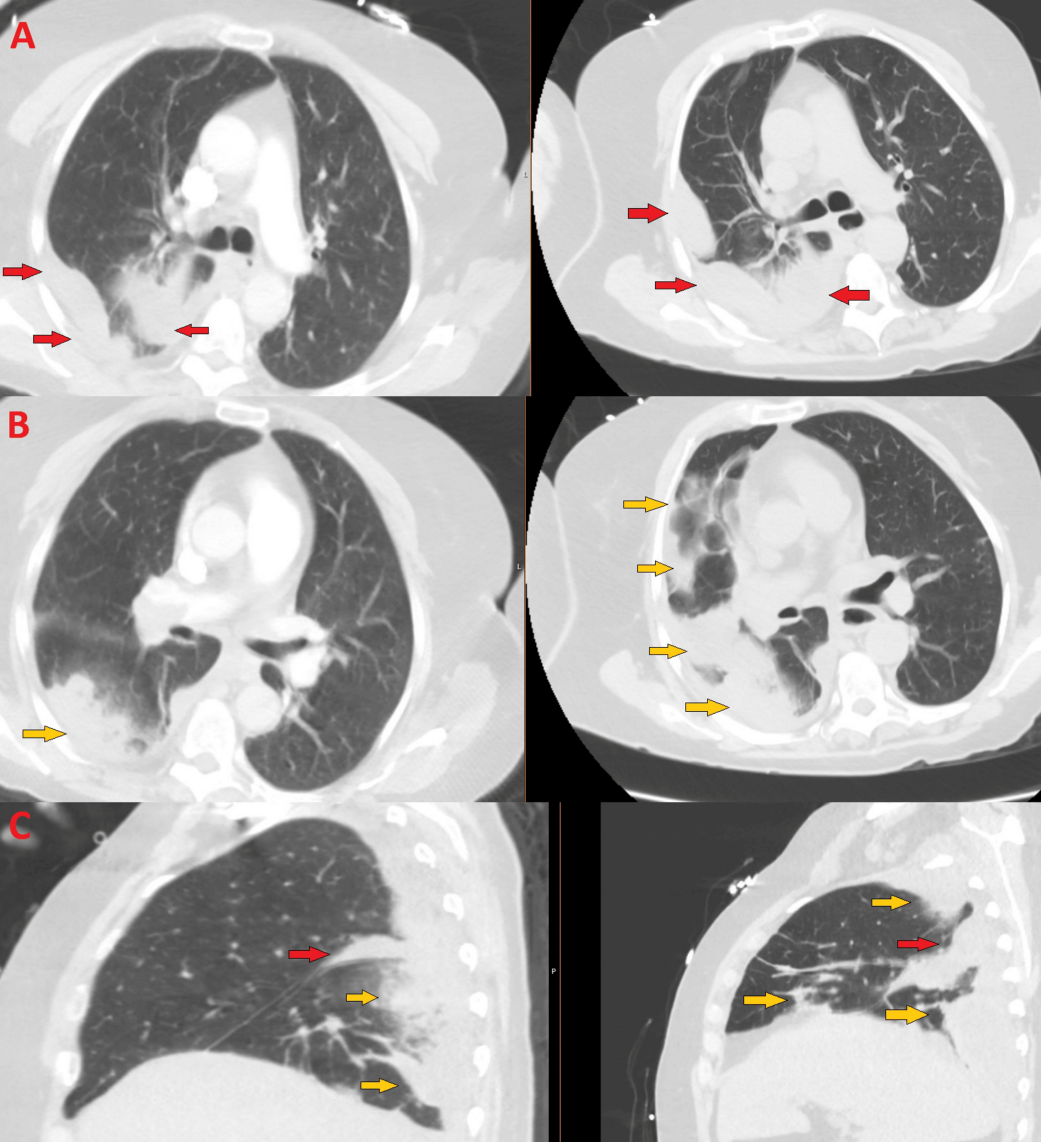
Worsening pulmonary infarct on rapid response Comparison of axial cuts (A, B) and sagittal cut (C) of CTA Chest on initial ED imaging (left) vs rapid response (right) demonstrating worsening pulmonary infarct (yellow arrows), stable/slightly worsening small pleural effusions (red arrows). Formal radiologist interpretation: "Redemonstrated right main pulmonary trunk and right lower lobe segmental pulmonary emboli. No evidence of right heart strain. Evolving right lower lobe pulmonary infarct. Right pleural effusion, increased compared to prior."

On the third day of hospitalization, the patient had a rapid response called for worsening hypoxemia and respiratory distress, requiring escalation to the intensive care unit and high flow nasal cannula (HFNC) of 30L flow rate and 80% FiO2. An arterial blood gas (ABG) was done on these settings which showed an arterial partial pressure of oxygenation (PaO2) of 71mmHg. She remained hemodynamically stable, and a point of care cardiac ultrasound was again, normal. A third CTA chest was performed showing stable pulmonary embolism but worsening consolidations and pleural effusions (Figure 3). Evaluation for thrombectomy was recommended at this time for refractory hypoxia and worsening pulmonary infarct.

**Figure 4:**
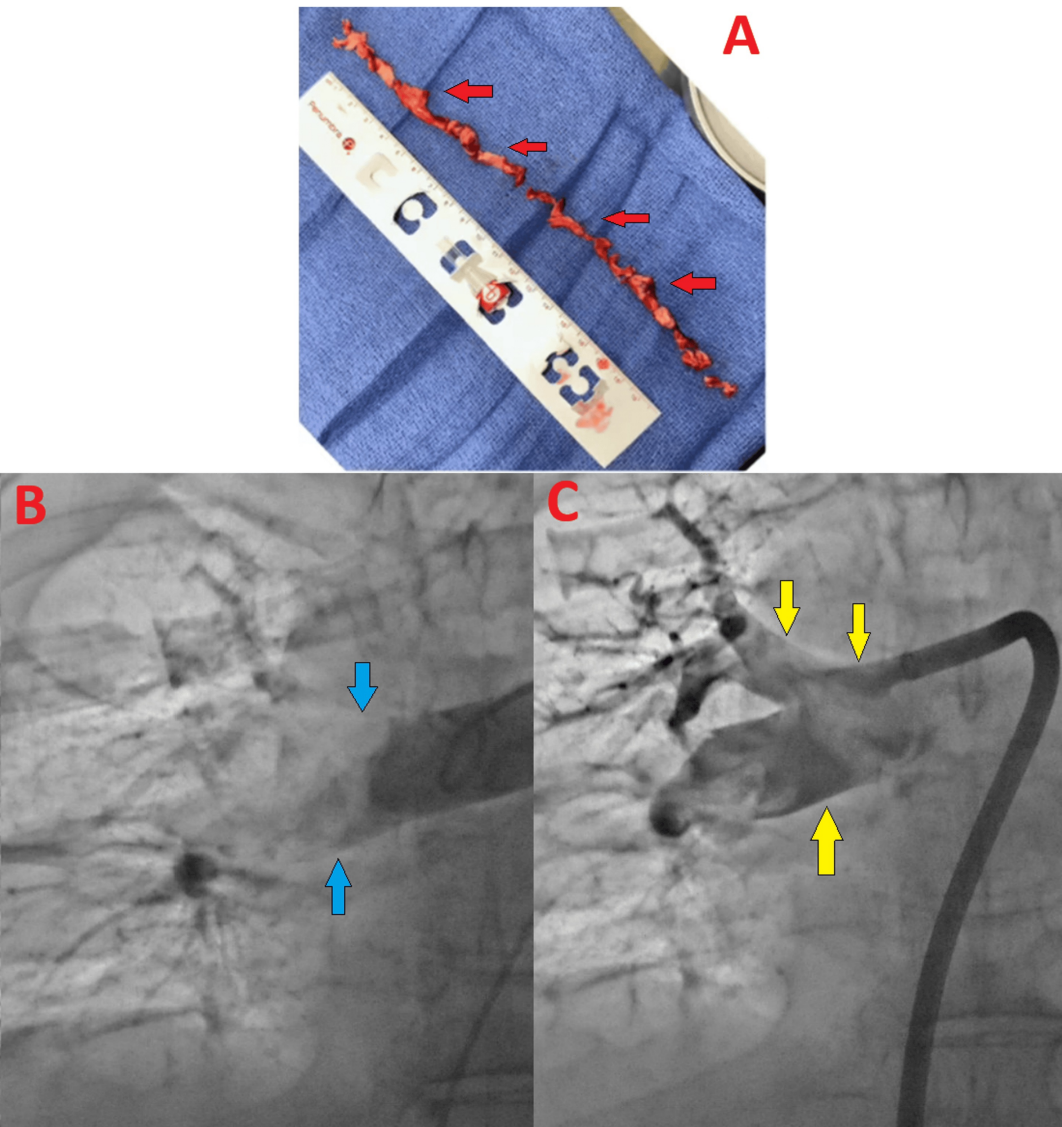
Aspiration thrombectomy Thrombus removed with aspiration thrombectomy (A - top, red arrows). Angiogram showing no flow in the right pulmonary artery (B - bottom left, blue arrows). Restoration of flow after aspiration thrombectomy (C - bottom right, yellow arrows).

On day 4 of hospitalization, the patient was taken to the catheterization laboratory and the right femoral vein was accessed for right heart catheterization. Initial hemodynamics was measured: Right Atrium 13mmHg, Right Ventricle 41/12mmHg, Pulmonary Artery 40/20mmHg (mean 29mmHg), Pulmonary Artery Saturation 47.8%, Cardiac Output 3.58L/min, Cardiac Index 1.8L/min/m^2^, and Pulmonary Artery Pulsatility Index 1.5. A bilateral pulmonary angiogram was performed revealing a large occlusive embolus in the right main pulmonary artery. Aspiration thrombectomy (Indigo System, Penumbra, Inc., USA) was pursued yielding significant clot burden (Figure 4). Subsequent pulmonary artery pressures improved to 37/15mmHg (mean 24mmHg).

The patient was weaned to 4L NC hours after the procedure and within 48 hours was weaned completely off oxygen. She was subsequently discharged on apixaban.

## Discussion

Pulmonary infarction after pulmonary embolism is relatively common and estimated to occur in approximately 30% of patients and possibly up to 70% as found on autopsies [5]. Treatment is directed at the underlying etiology, the pulmonary embolism, and thus anti-coagulation and supportive care are standard. Oxygen therapy may be indicated temporarily and, those who can be discharged have no significant adverse impairments at three months [6], near complete restoration of pulmonary perfusion at one year [7]. Yet, Kaptein et al. [6] describe symptomatic pulmonary infarction in those with more proximal PE, which was consistent in our patient with extensive right pulmonary artery occlusion starting just distal to the bifurcation of the main pulmonary artery. Our patient was initially discharged after her first hospitalization for PE on room air. Despite a slight improvement in the PE on her second CTA chest, she developed progressive hypoxemia and increasing pulmonary infarction, prompting consideration for more aggressive therapeutics.

Selection criteria for catheter-directed therapy for pulmonary embolism remain challenging with ongoing controversy, especially in the treatment of intermediate-risk PE. In this population, it is difficult to predict who would benefit. Long-term benefits including mortality reduction of CDT are also unclear. The European Society of Cardiology (ESC) recommends CDT be considered in patients with high-risk PE that have contraindications for thrombolysis or as rescue therapy when first-line treatments have failed [2]. Recent updates to the CHEST guidelines recommend CDT only in patients with high-risk PE with high bleeding risk, failed systemic thrombolysis, or rapid progressive hemodynamic collapse only if expertise and resources are available [8]. The American Heart Association (AHA) guidelines recommend the use of CDT for intermediate-risk PE for those only with clinical deterioration (not specifically defined) after therapeutic anti-coagulation [9]. There are no firm recommendations for intermediate-risk PE amongst these major guidelines.

The European Society of Cardiology subdivides intermediate-risk PE into intermediate-high and intermediate-low risk. The former requires both RV dysfunction and elevated cardiac troponin levels, and the latter only one or no positive criteria [2]. Our patient would be categorized into the intermediate-low risk with no elevated cardiac markers and no RV dysfunction. To date, there is a rarity of case reports that describe CDT in those with no RV dysfunction or categorized with intermediate-low risk PE. He and Liu published a case in which their patient had a vasculitis associated with intermediate-risk pulmonary embolism. Like our case, the patient was initially discharged on anti-coagulation, had subsequent imaging demonstrating little to no improvement, and CDT was utilized with good outcome [10]. Similar to our patient, there were no signs of RV dysfunction however the decision to pursue CDT in this case was on routine follow-up, a major difference from our patient that required re-admission for progressive deterioration and refractory hypoxemia.

While guidelines describe progressive hemodynamic deterioration as a trigger to pursue CDT, our patient did not exhibit this. Our patient was hemodynamically stable throughout her entire hospitalization and had an echocardiogram demonstrating no right heart strain. Furthermore, no guideline specifically mentions refractory hypoxemia or progressive pulmonary infarct as a criterion. Yet, CDT was pursued due to her progression of disease. We suspected she would benefit due to her proximal occlusion and extent of pulmonary ischemia. And following CDT, the patient quickly reversed her clinical course and returned to baseline room air.

## Conclusions

Pulmonary embolism is commonly treated with anti-coagulation. It is unclear which intermediate-risk PE patients would benefit from more advanced therapy such as CDT. Although there are no current guidelines for using CDT in pulmonary infarct or progressive hypoxemia, our patient clearly benefited. Usual treatment is supportive, however, in cases of patients with worsening infarction or hypoxemia, CDT can be offered as a more aggressive modality. As catheter technology advances and user proficiency develops, there may be a role in more aggressive, definitive treatment.
